# Supplementary material for: Innovative Low-Cost Composite Nanoadsorbents Based on Eggshell Waste for Nickel Removal from Aqueous Media
Source: Nanomaterials (Basel). 2023 Sep 16;13(18):2572. doi: 10.3390/nano13182572 (PMC10537637; doi:10.3390/nano13182572)
Supplement: Supplementary file 1 [file nanomaterials-13-02572-s001.zip › nanomaterials-2594400-supplementary.pdf]

SUPPLEMENTARY INFORMATION

Innovative Low-Cost Composite Nanoadsorbents Based on Eggshell Waste for Nickel Removal from Aqueous Media

Adina-Elena Segneanu[1], Roxana Trusca[2], Claudiu Cepan[3], Maria Mihailescu[3,4], Cornelia Muntean[3,4], Daniel Dumitru Herea[5], Ioan Grozescu[3] and Athanasios Salifoglou[6,✉]

1 Institute for Advanced Environmental Research, West University of Timisoara (ICAM-WUT), 4 Oituz St., 300086 Timişoara, Romania
2 National Center for Micro and Nanomaterials, Politehnica University of Bucharest, Str. Splaiul Independenţei, Nr. 313, 060042 Bucharest, Romania
3 Department of Applied Chemistry and Engineering of Inorganic Compounds and the Environment, University Politehnica Timisoara, Piata Victoriei Nr. 2, 300006 Timisoara, Romania4 Research Institute for Renewable Energy, 138 Gavril Musicescu St., 300501 Timisoara, Romania
5 National Institute of Research and Development for Technical Physics, 47 Mangeron Blvd, 700050 Iasi, Romania
6 Laboratory of Inorganic Chemistry and Advanced Materials, School of Chemical Engineering, Aristotle University of Thessaloniki, 54124 Thessaloniki, Greece
✉ Author to whom correspondence should be addressed

Author E-mail addresses: adina.segneanu@e-uvt.ro; truscaroxana@yahoo.com, cepanclaudiu@gmail.com; mihailescumia@gmail.com; cornelia.muntean@upt.ro; dherea@phys-iasi.ro; ioangrozescu@gmail.com; salif@auth.gr.

FIGURES

Figure S1: Nitrogen adsorption-desorption isotherms for FEZ adsorbent, EZ, eggshell, and zeolite.

Figure S2: FT- IR spectra of zeolite (**A**), eggshell (**B**), EZ (**C**), and FEZ (**D**).

Figure S3: EDX spectra of zeolite (**A**), and eggshell (**B**).

Figure S4: SEM Live map (**1**) and EDX spectra showing the distribution of elements (**2**) in EZ (**A**), and FEZ (**B**).

Figure S5: XRD patterns of zeolite (**A**), eggshell (**B**), EZ (**C**), and FEZ (**D**).

Figure S6: Plot of the experimental data using the linearized form of (**A**) Langmuir model, and (**B**) Freundlich model.

Figure S7: Van't Hoff plot for nickel adsorption on FEZ and EZ.

Figure S8: (**A**) Pseudo-first-order model fitting diagram, (**B**) Pseudo-second-order model fitting diagram, and (**C**) Intraparticle diffusion model fitting diagram.

Figure S9: FT-IR spectra of EZ (**A**) after adsorption, and (**B**) before and after adsorption.

Figure S10: FT-IR spectra of FEZ (**A**) after adsorption, and (**B**) before and after adsorption.

Figure S11: SEM micrographs of EZ (**A, 30 µm; C, 50 µm**) before adsorption, and (**B, 30 µm; D, 50 µm**) after adsorption.

Figure S12: SEM micrographs of FEZ (**A, 30 µm; C, 50 µm**) before adsorption, and (**B, 30 µm; D-E, 50 µm**) after adsorption.

Figure S13: Live maps of EZ (**A**), and FEZ (**B**) after adsorption.

Figure S14: EDX spectrum of EZ (**A**), and FEZ (**B**) after adsorption.

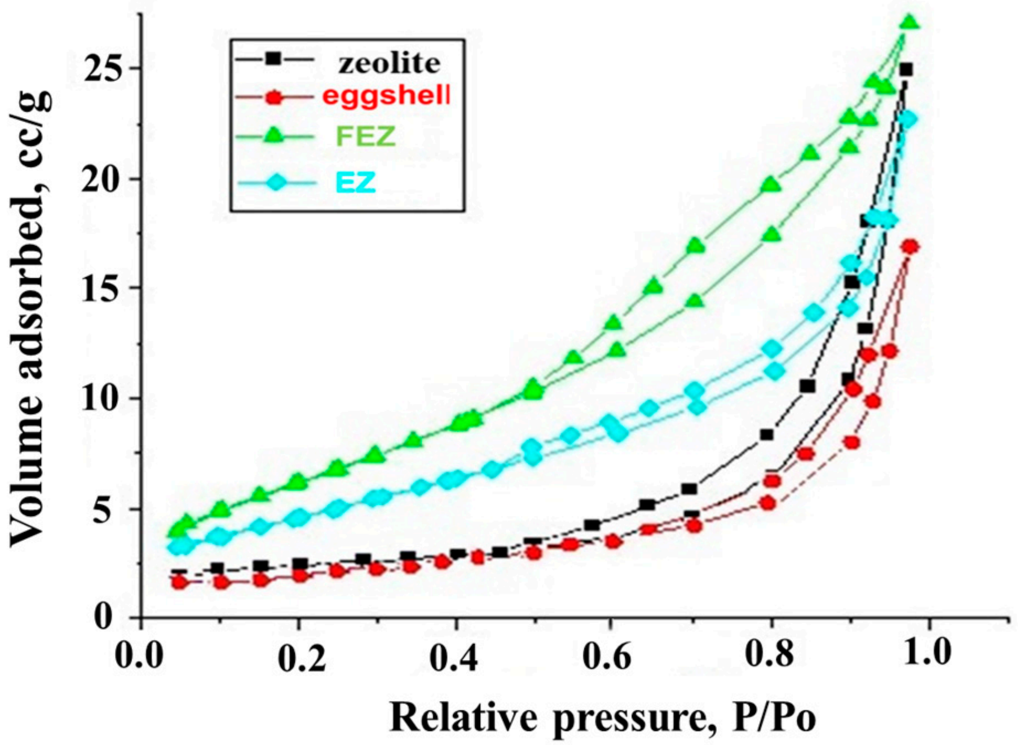
zeolite
eggshell
FEZ
EZ
Volume adsorbed, cc/g
0
5
10
15
20
25
0.0
0.2
0.4
0.6
0.8
1.0
Relative pressure, P/Po

Figure S1

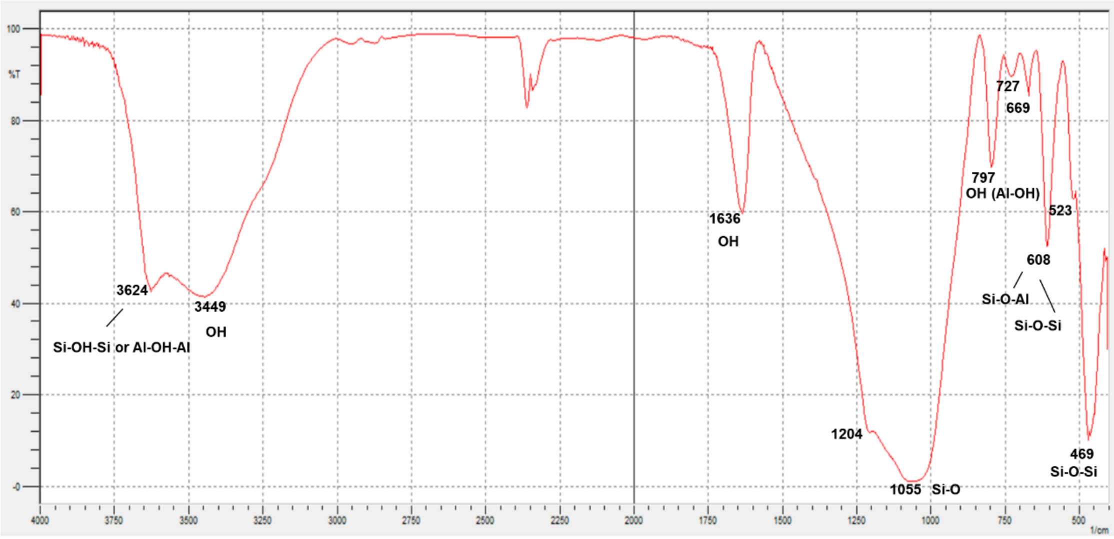

Figure S2A

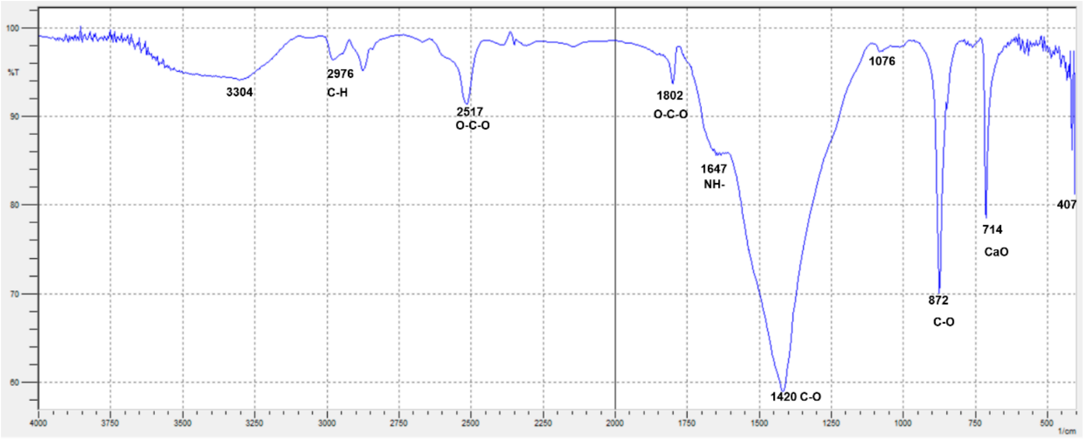

Figure S2B

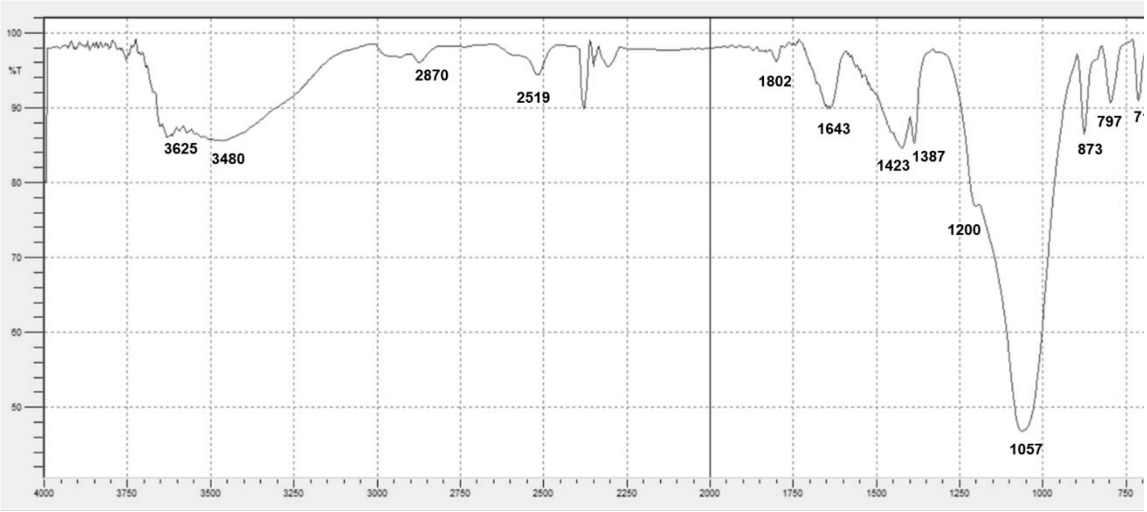

Figure S2C

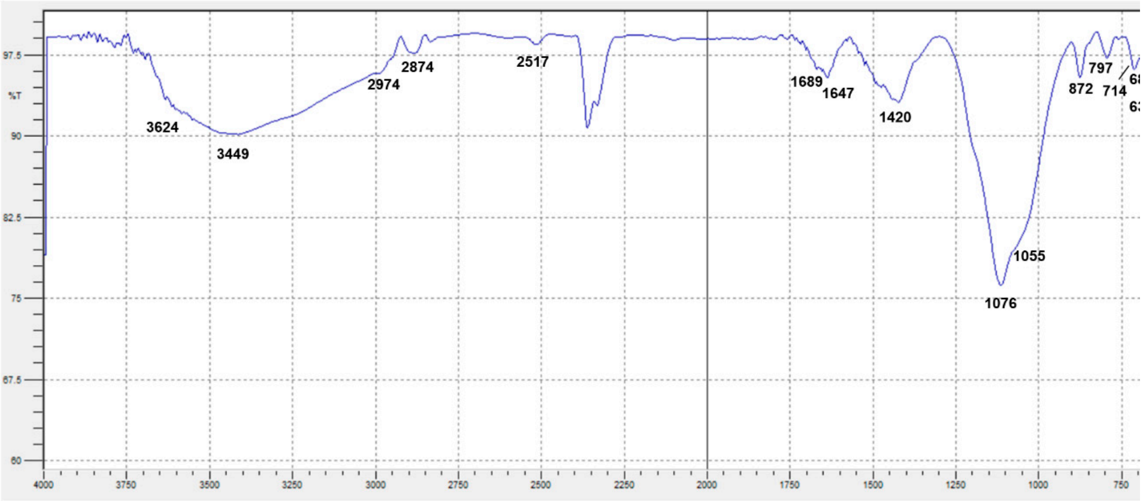
97.5
%T
90
82.5
75
67.5
60
4000
3750
3500
3250
3000
2750
2500
2250
2000
1750
1500
1250
1000
750
3624
3449
2974
2874
2517
1689
1647
1420
1076
1055
872
797
714

Figure S2D

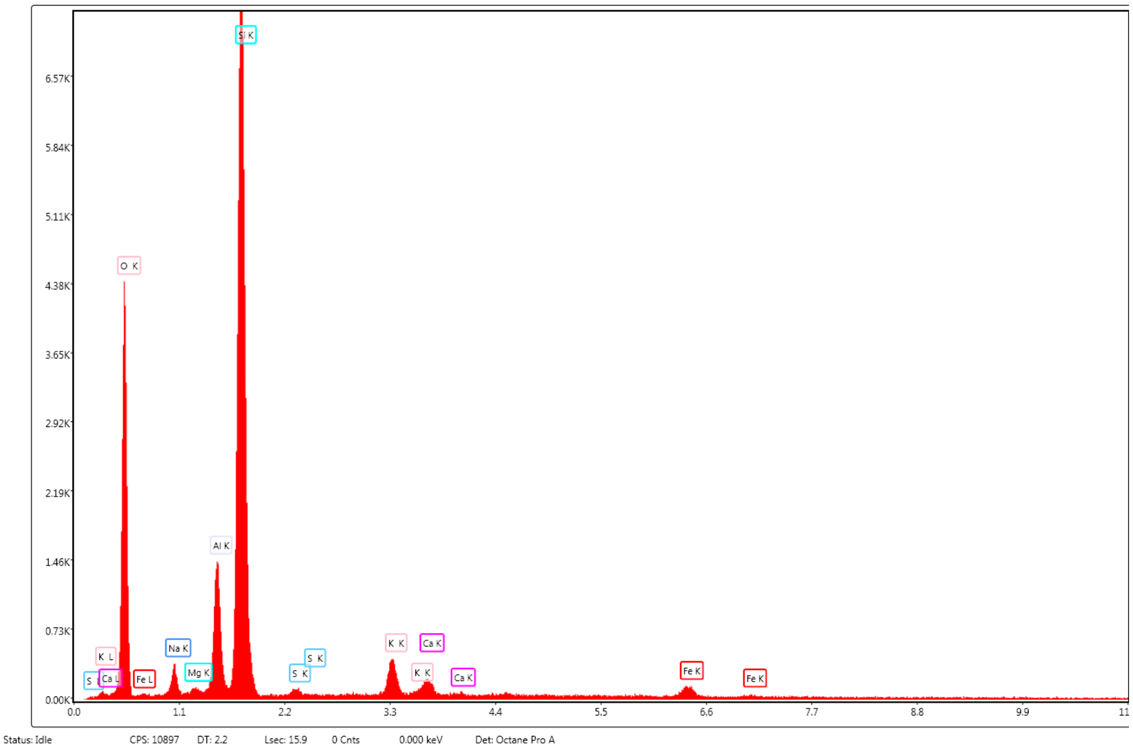

Figure S3A

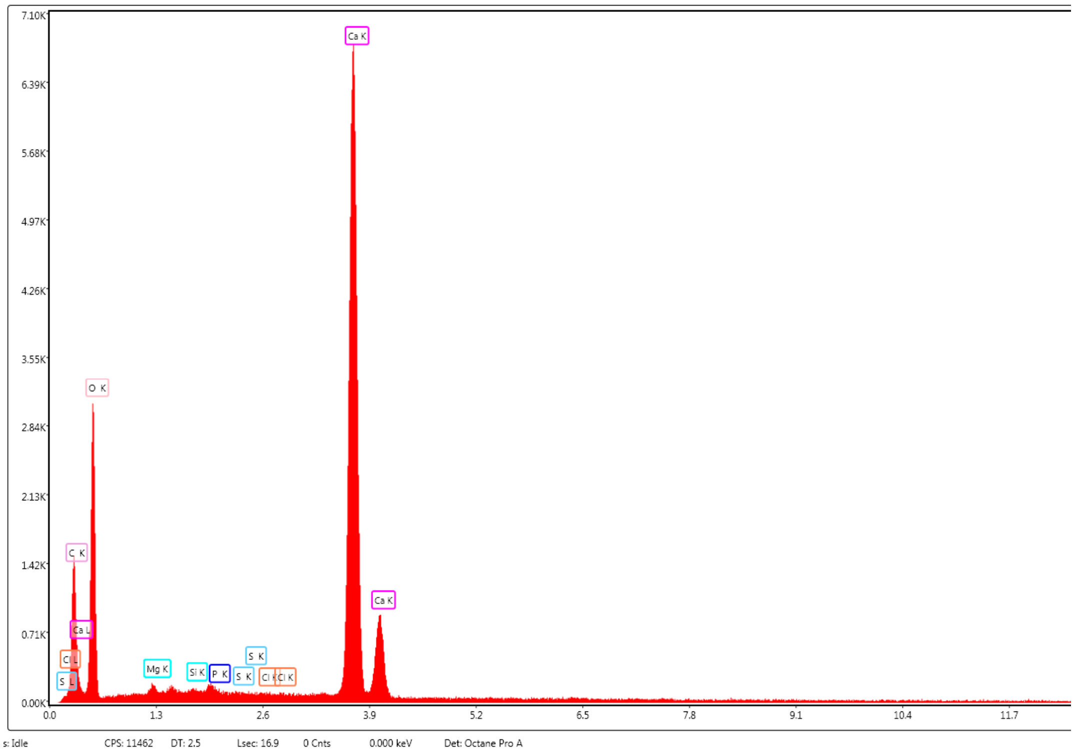

Figure S3B

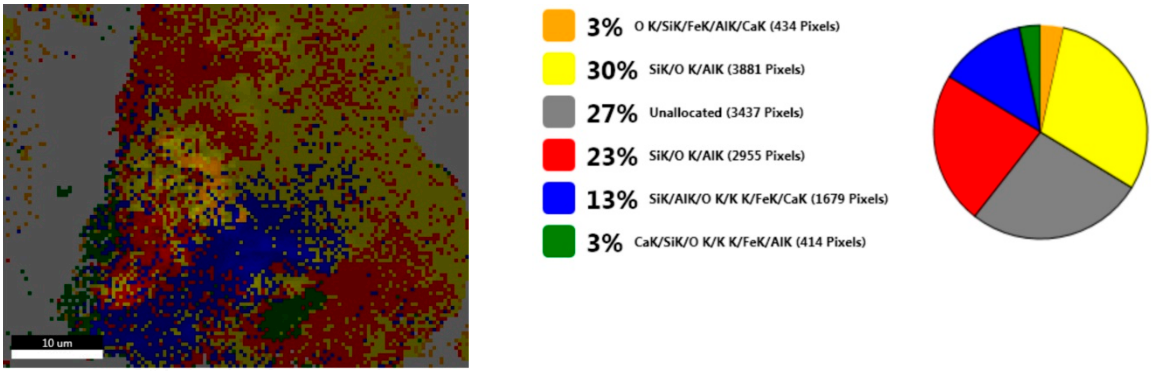

Figure S4A(1)

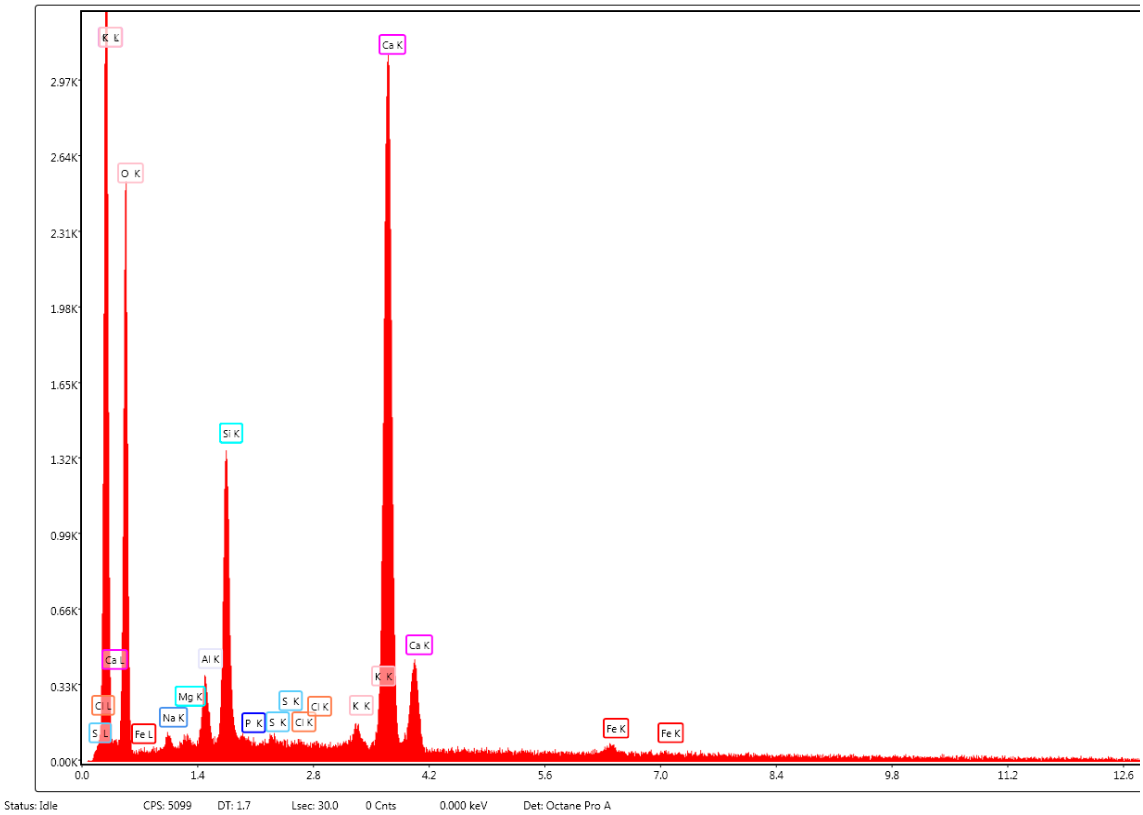

Figure S4A(2)

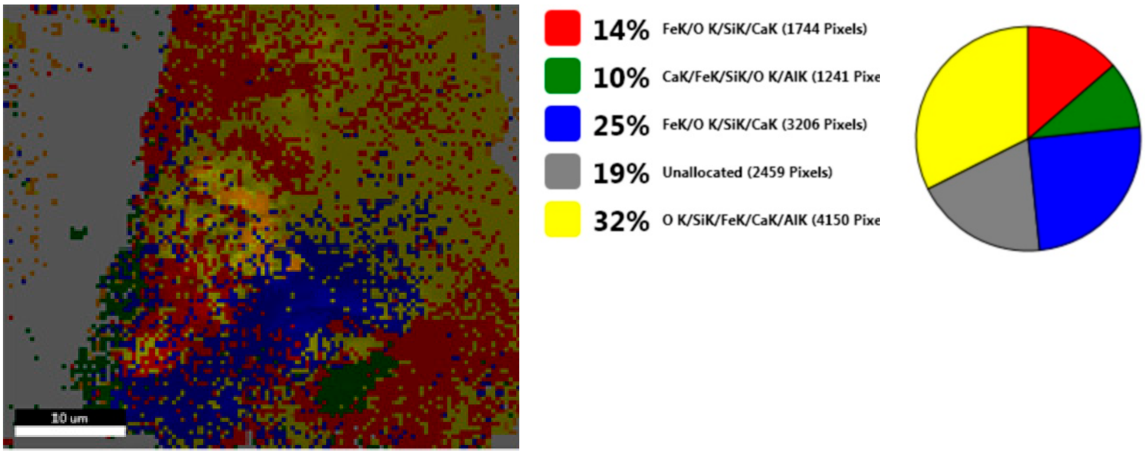

Figure S4B(1)

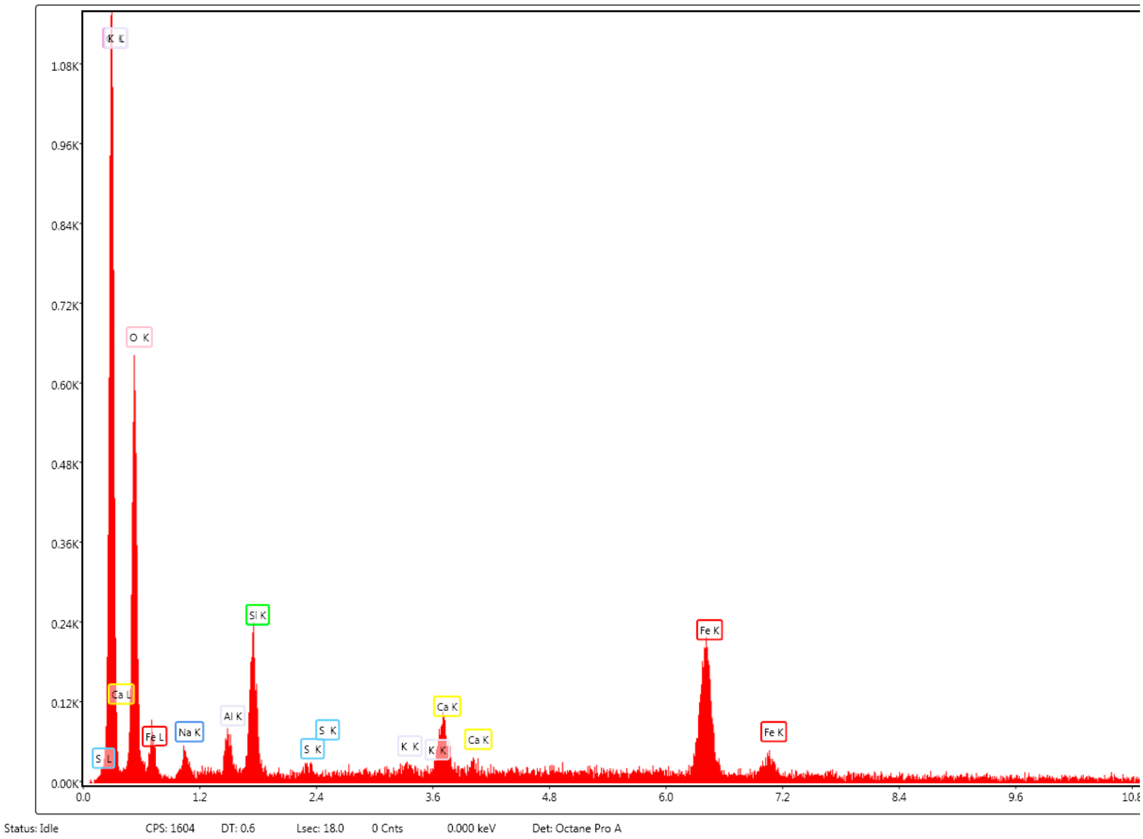

Figure S4B(2)

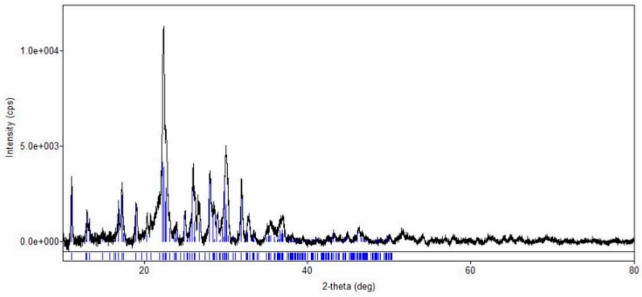

A

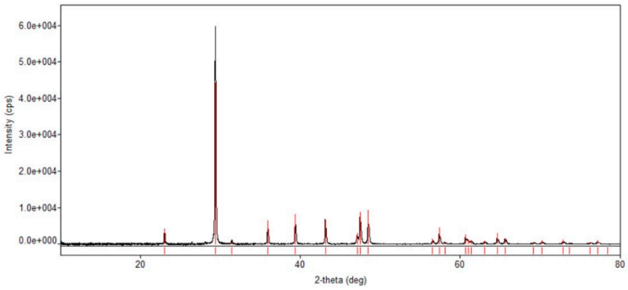

B

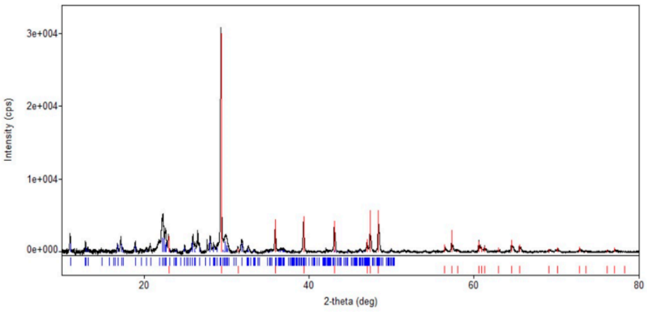

C

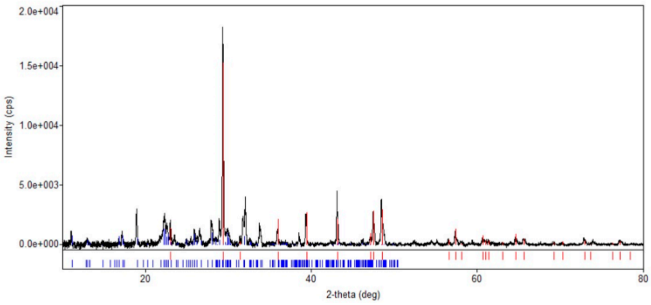

D

Figure S5

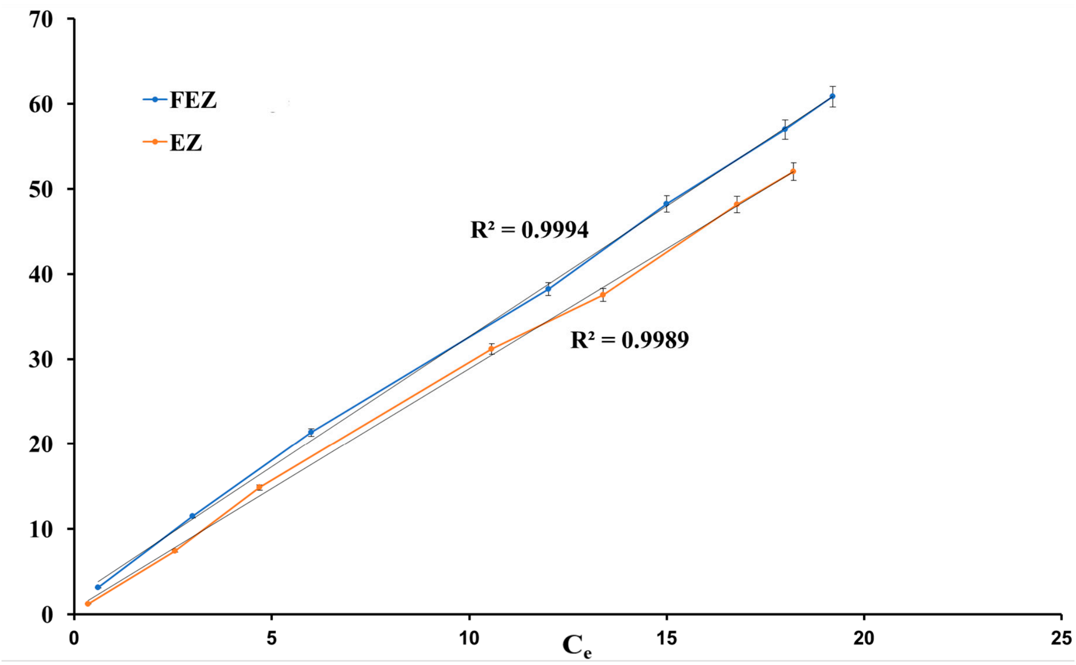
70
60
50
40
30
20
10
0
FEZ
EZ
R² = 0.9994
R² = 0.9989
0
5
10
15
20
25
Ce

A

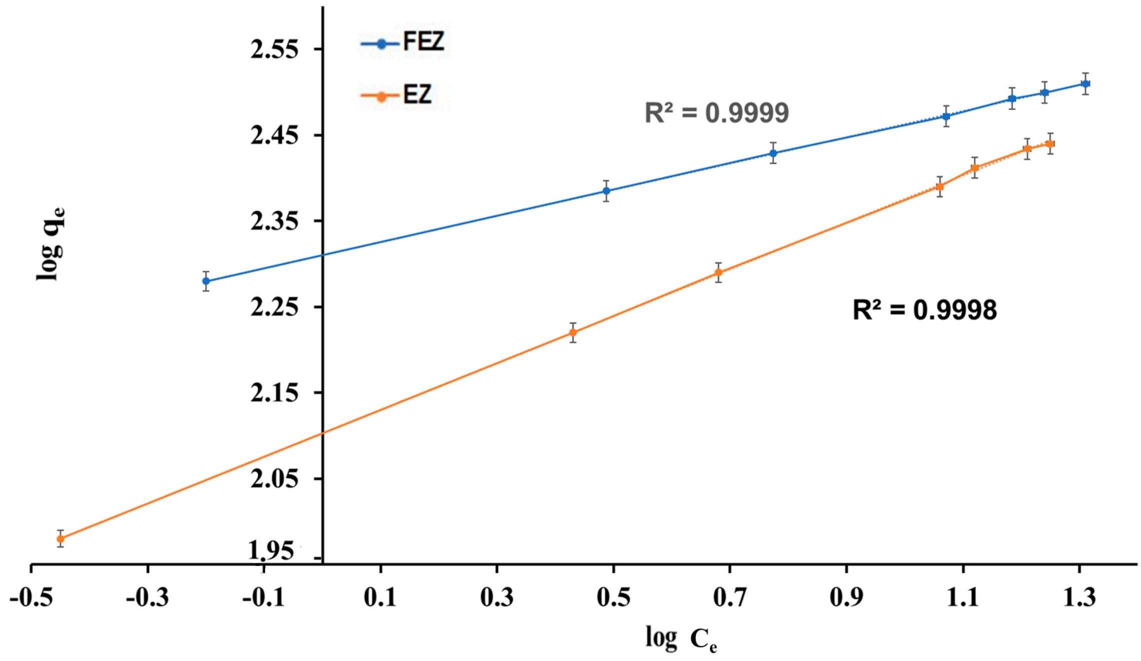
FEZ
EZ
R² = 0.9999
R² = 0.9998
2.55
2.45
2.35
2.25
2.15
2.05
1.95
log qe
-0.5
-0.3
-0.1
0.1
0.3
0.5
0.7
0.9
1.1
1.3
log Ce

B

Figure S6

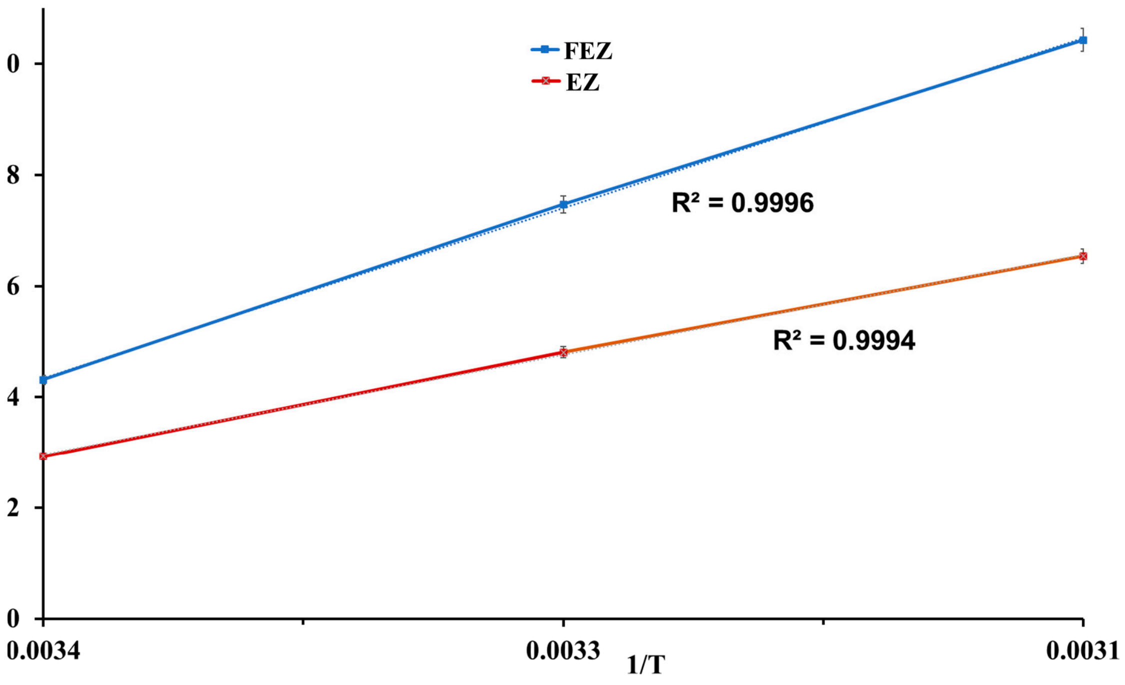

Figure S7

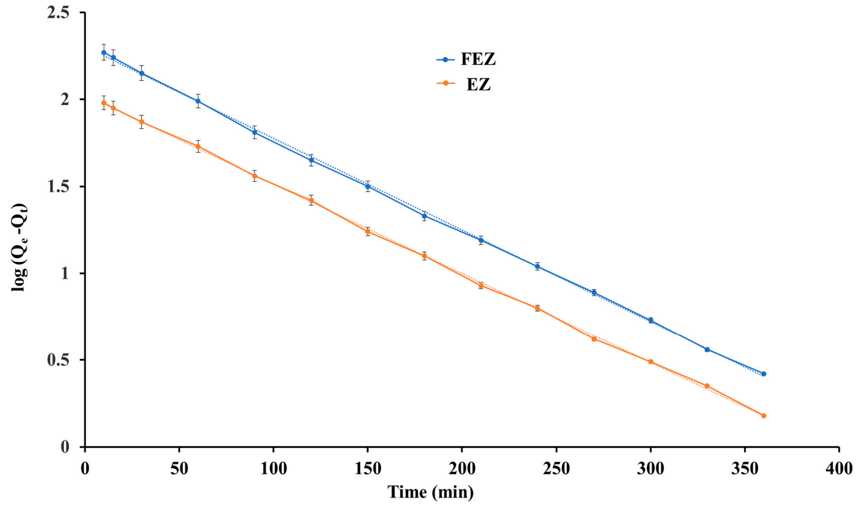

A

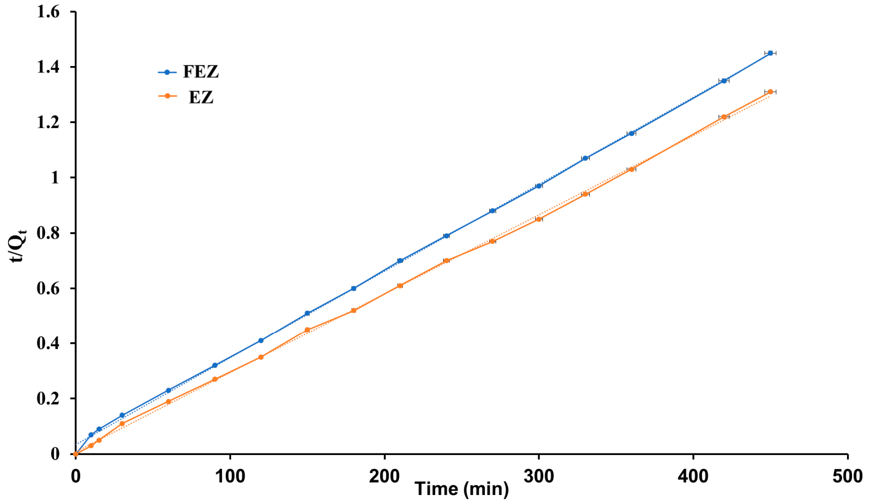

B

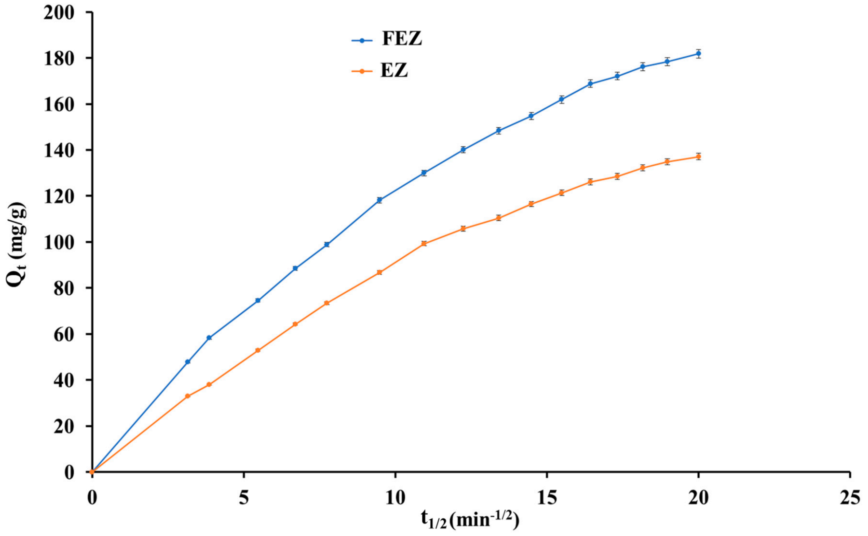

C

Figure S8

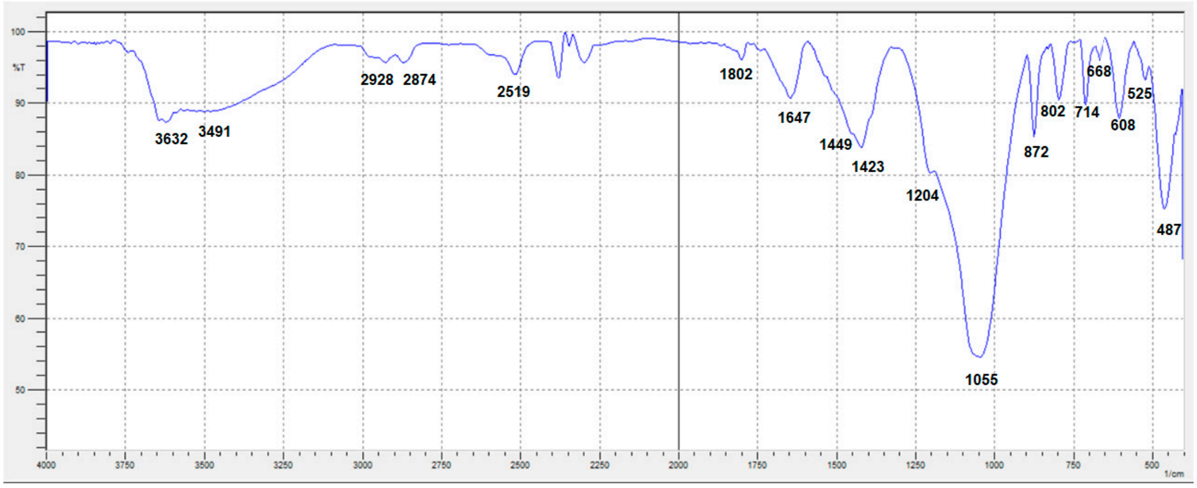

Figure S9A

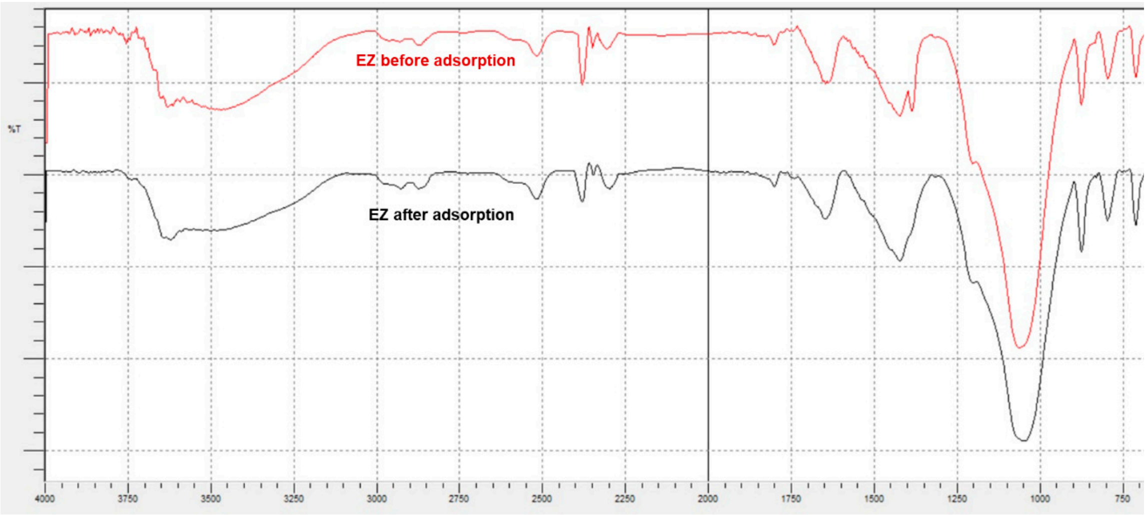

Figure S9B

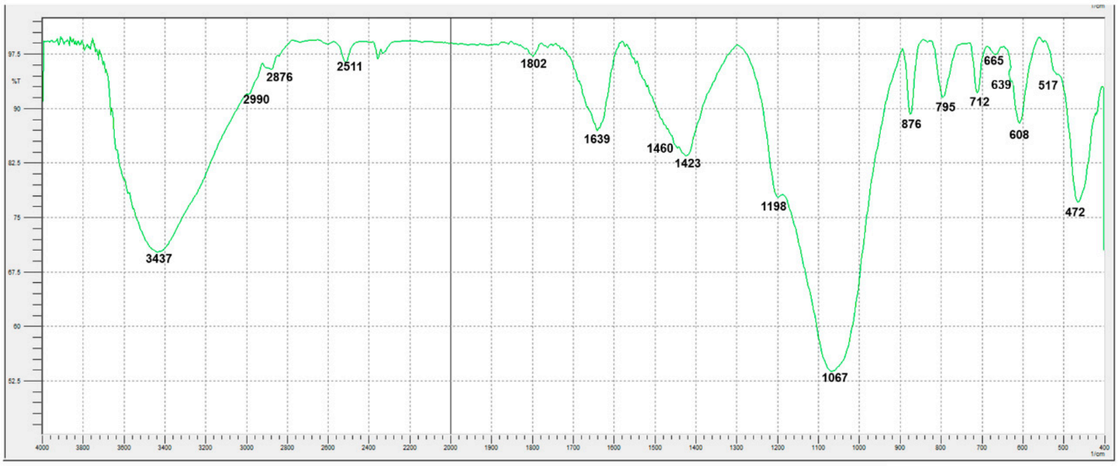

Figure S10A

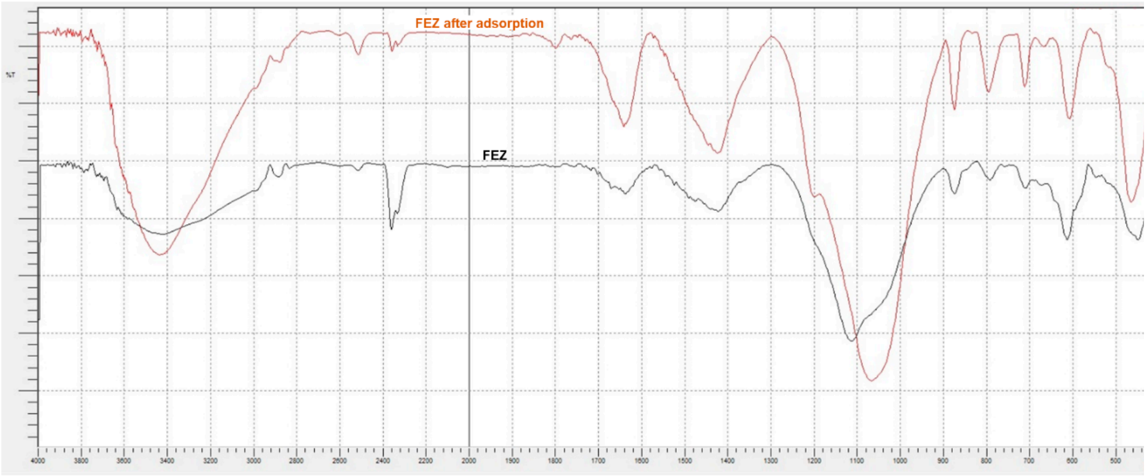

Figure S10B

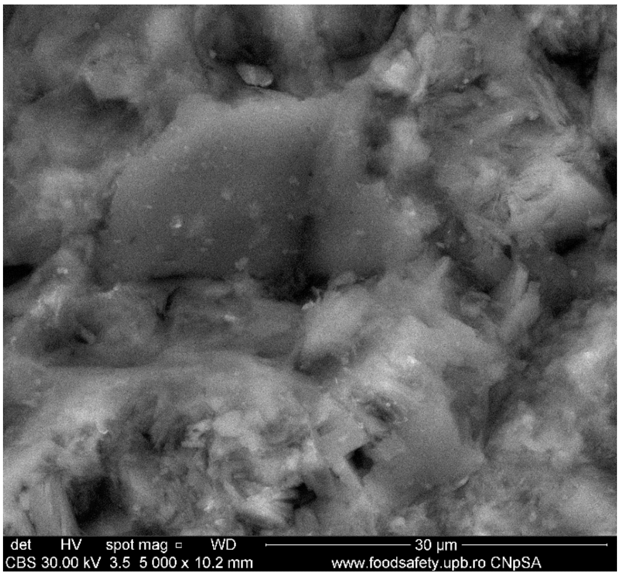

A

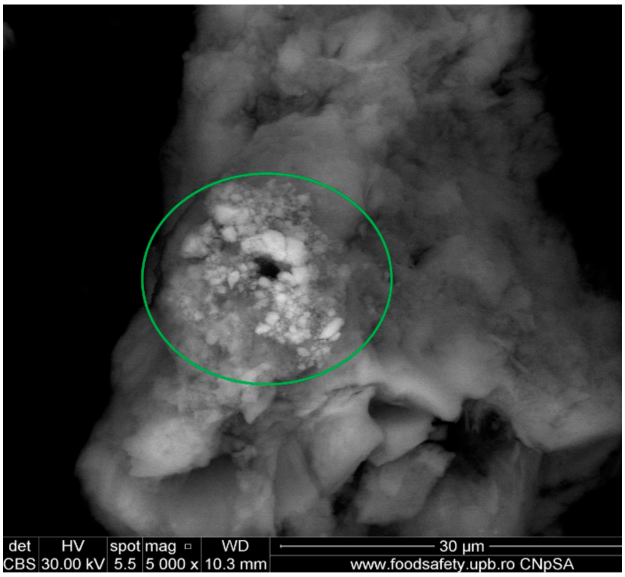

B

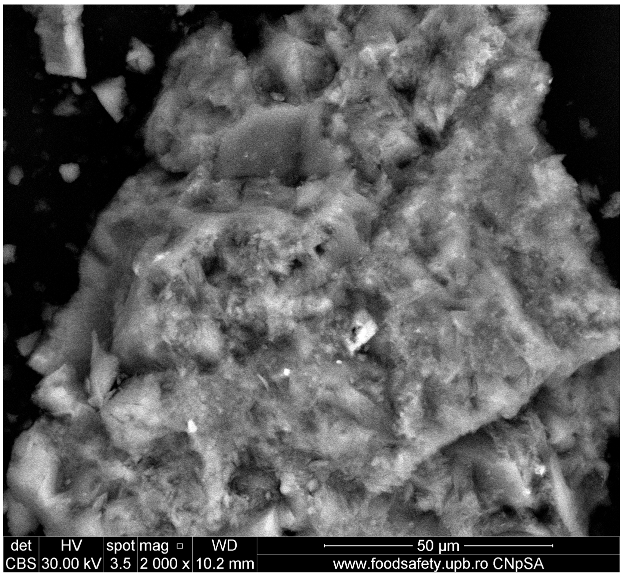

C

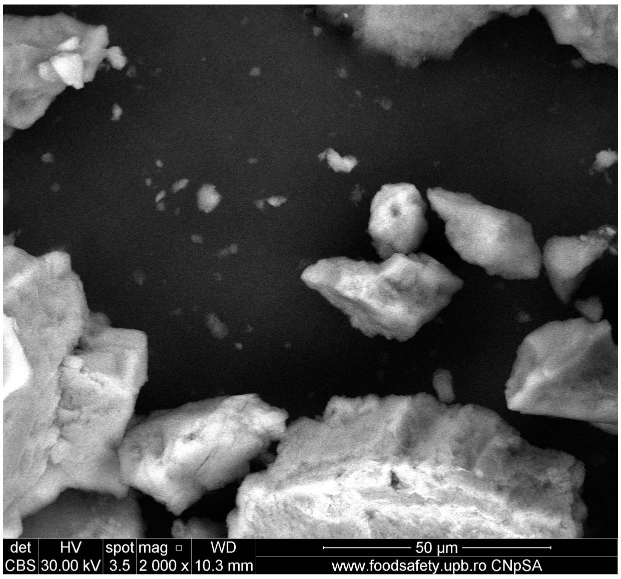

D

Figure S11

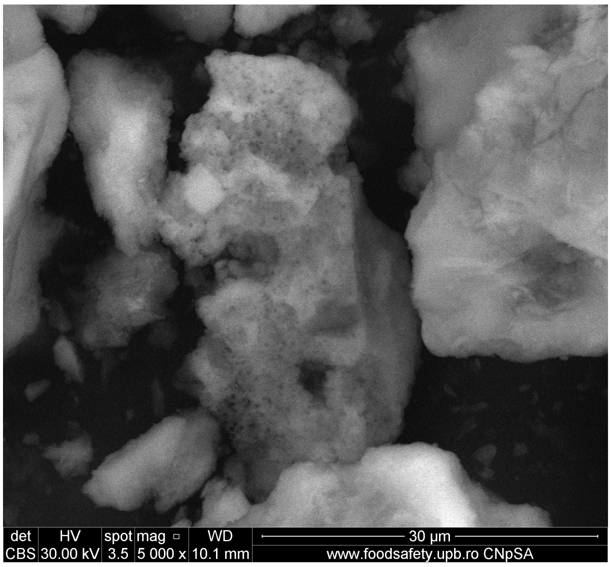

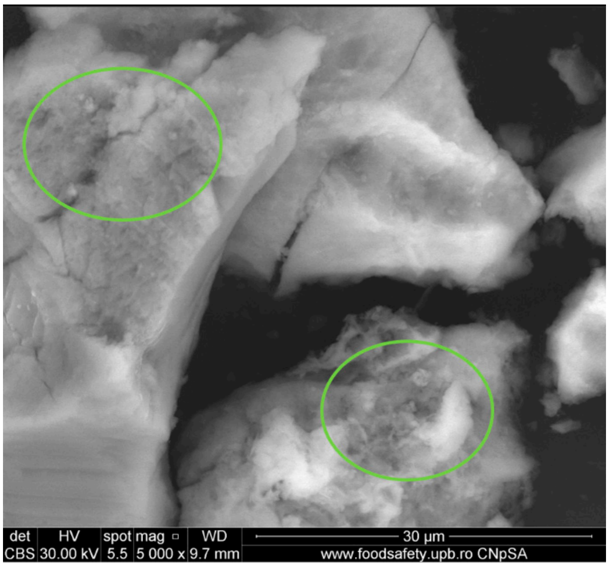

A **B**

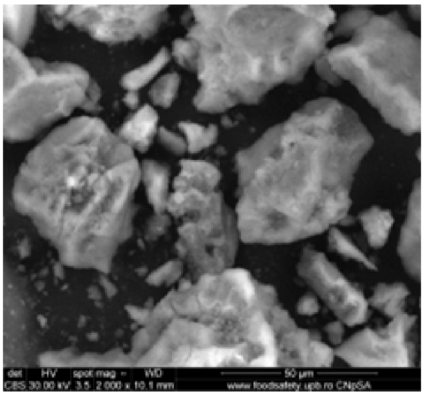

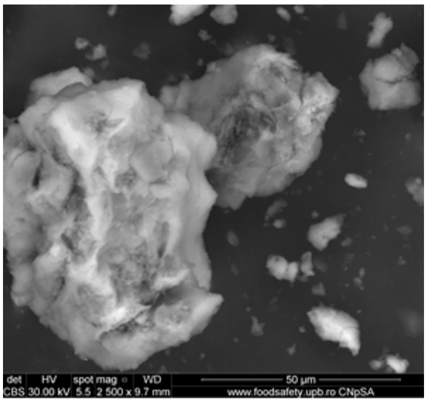

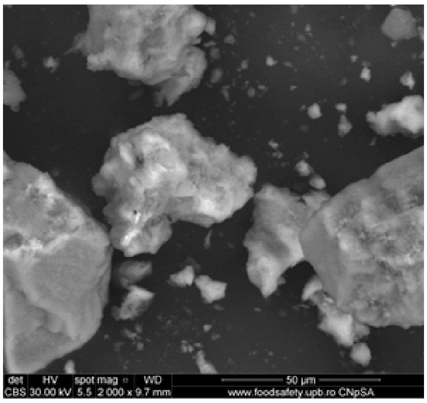

C **D** **E**

Figure S12

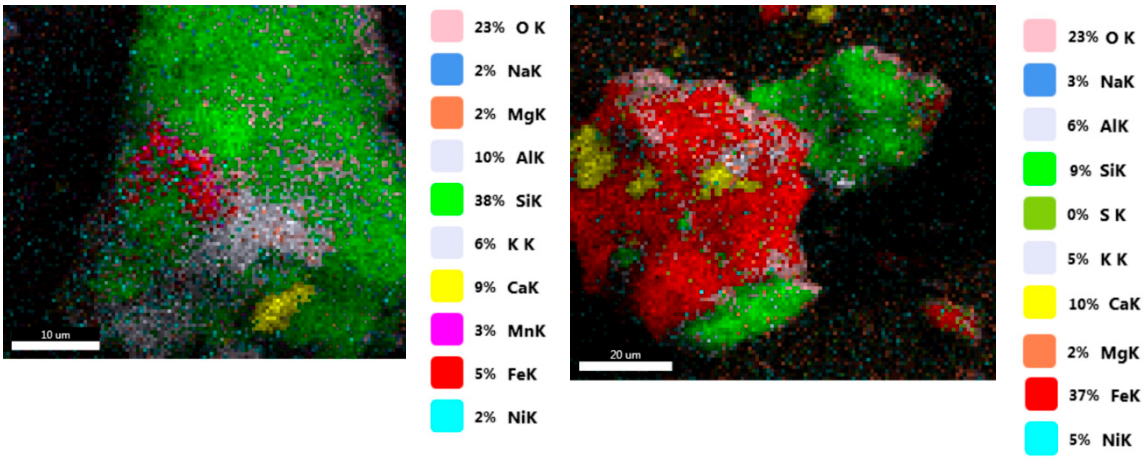

A **B**

Figure S13

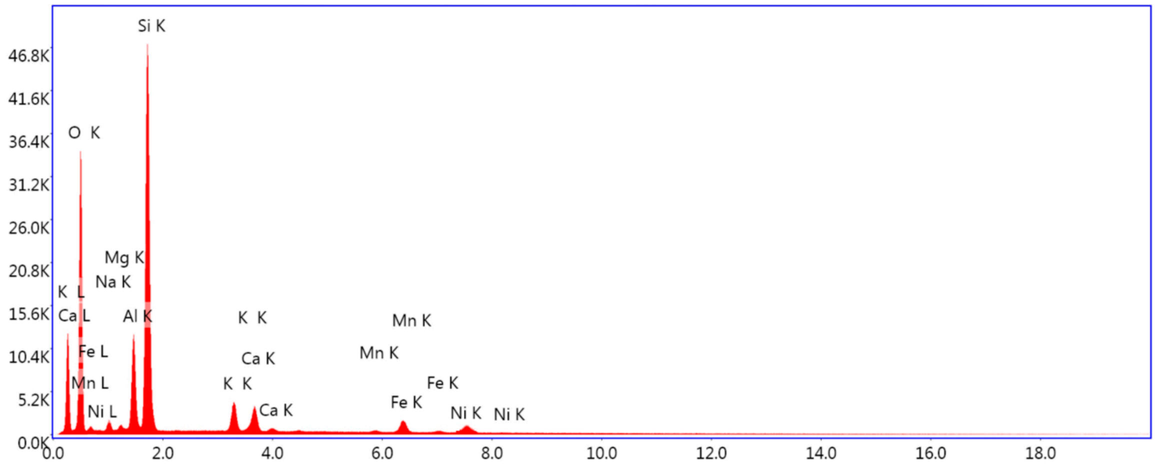

Figure S14A

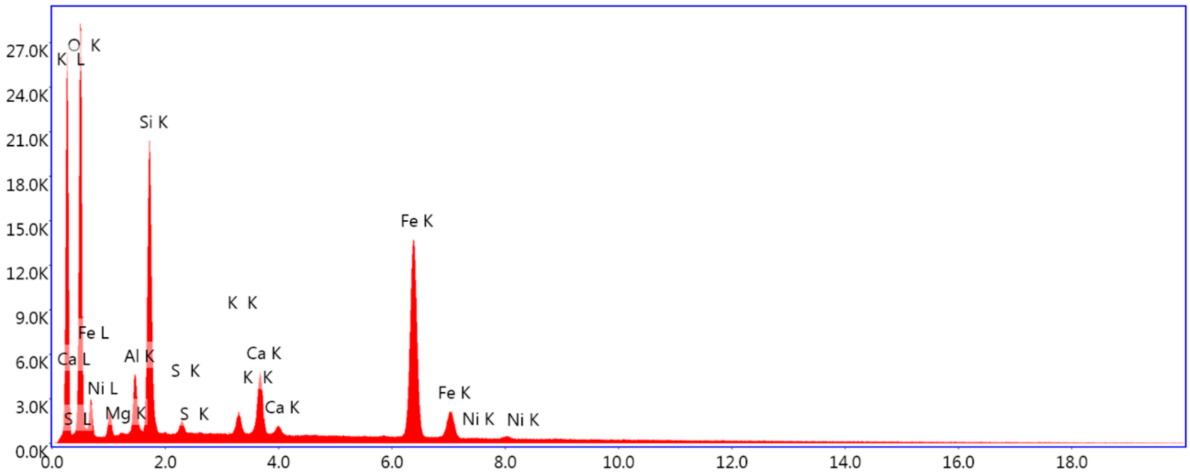

Figure S14B
